# What motivates informal caregivers of people with dementia (PWD): a qualitative study

**DOI:** 10.1186/s12904-019-0491-9

**Published:** 2019-11-28

**Authors:** Shakiba Zahed, Maryam Emami, Shahrzad Bazargan-Hejazi, Ahmad Ali Eslami, Majid Barekatain, Fereshteh Zamani-Alavijeh

**Affiliations:** 1 Candidate in Health Education and Health Promotion, Student Research Committee, School of Health, Isfahan University of Medical Sciences, Isfahan, Iran; 2 Department of Psychiatry, School of Medicine, Isfahan University of Medical Sciences, Isfahan, Iran; 3 Department of Psychiatry, College of Medicine, Charles Drew University of Medicine and Science, and David Geffen School of Medicine, the University of California at Los Angeles (UCLA), Los Angeles, USA; 4 Department of Health Education and Promotion, School of Health, Isfahan University of Medical Sciences, Isfahan, Iran; 5 Department of Psychiatry, School of Medicine, Isfahan University of Medical Sciences, Isfahan, Iran; 6 Department of Health Education and Promotion, School of Health, Isfahan University of Medical Sciences, Isfahan, Iran

**Keywords:** Alzheimer’s disease and other dementias, Caregivers, Motivation, Religious, And spiritual motivators

## Abstract

**Background:**

The burden of caring for People with Dementia (PWD) is heavy; identifying incentives that motivate them in providing care is essential in facilitating and optimizing care. This study aims to explore and describe these motivating factors.

**Methods:**

We conducted this qualitative study between January 2016 and January 2017 in Isfahan, Iran. Data were extracted through in-depth, semi-structured interviews with 19 caregivers of PWD. These data were then examined through thematic content analysis.

**Results:**

We identified four categories of psychological motives based on the caregivers’ feedback and experience. These include 1) Moral-based motives, 2) Religious, and spiritual motives; 3) Financial motives, and 4) Wicked motives.

**Conclusions:**

Our results revealed several aspects of caregivers’ motives. They include moral, religious, and spiritual aspects; sharing housing accommodations, and the likelihood of inheriting a portion of the patient’s assets based on unspoken rules and informal arrangements in the family, and wicked and immoral aspects. These findings can inform future efforts in enhancing the experiences of caregivers of PWD, and subsequently, the quality of care these patients receive. It further suggests that family members, members of a religious and spiritual organization, as well as social media, could play important roles in setting the stage.

## Background

Globally, 47.5 million people were diagnosed with dementia in 2015, and this figure is expected to reach 75.6 million in 2030, and over 135 million by 2050 [1]. This implies globally 7.7 million new cases or one new case every 4 seconds requires care and attention from the system, family members, or other sources, thus, challenging the financial, physical, and psychological resources in the countries including in Iran [2, 3].

With the higher prevalence of People with Dementia (PWD), identifying a source of regular caregiving for PWD has become a routine practice in Iran and other countries [2, 4–6]. Family members (informal caregivers), often assume this responsibility and serve as an interface between the PWD and health services [7, 8]. However, the responsibility of caregiving is also taken up by formal caregivers who often are paid for their services [9]. It is reported that informal caregivers are more likely motivated by emotional ties, cultural, spiritual, and religious responsibilities [2], while financial incentives motivate the formal caregivers [9].

Nevertheless, it is reported that the caregivers’ motivations influence their subjective experience of this role and, consequently the patterns of caregiving behavior [10–13]. Having higher positive intrinsic motivations have been associated with positive, meaningful caregiving experiences [11, 13–15], and consequently associated with higher quality care outcomes for the care receivers [16].

Formal and informal caregivers play an essential role in the provision of care for PWD. Exploring their perspectives on what motivates them to perform their responsibilities fully elucidates approaches to support them in providing optimal care [7, 17].

However, many of the caregivers’ beliefs, perceptions, and experiences are difficult to quantify [18] because they are influenced by a varying degree of socio-cultural and traditional values, which may deeply influence the individual’s subjective perception of these values [2, 18]. This is especially the case in a country such as Iran, where tradition, custom, and religious faith dominates virtually every facet of caregiving [2, 18].

In this study, we attempt to answer the following research question; what are the informal caregiver’s motives for taking up this role? We aim to explore and describe these motives. Once we better understand the caregivers’ motives for caring for the PWD, we can develop/tailor interventions to protect the caregivers’ altruistic motivation and diminish negative aspects of informal caregiving [19].

## Methods

### Study design, setting, and recruitment

We adopted a qualitative exploratory research approach. This approach can help to explore and better understand the process of engaging in a behavior and experiencing it from the perspective of the participant [20–22].

The study was conducted in Isfahan, Iran, between January 2016 and February 2017. Recruitment sites include the patient’s home and physician offices. We used convenient purposive sampling, and a nonprobability method used when researchers seek participants with specific characteristics that set them apart from others (for example husbands/wives, daughters/sons/ granddaughters/grandsons, friends, relatives, or no relationship) [23]. We also considered data saturation as a point of “diminishing returns” in a qualitative sample, whereby no new or relevant information emerges, or when the addition of more data does not add to the information already collected. Following these methods in qualitative studies allows for richer analytic generalizations [14, 24].

Female and male informal caregivers, 18 years of age and older who had at least 6 months of caregiving experience with PWD were eligible to participate in the study. This was determined by the study investigators (SZ), who subsequently enrolled 19 participants in the study and obtained informed consent.

### Data collection

Based on the prior arrangements with participants, the 19 in-depth, semi-structured interviews were conducted in Farsi (the predominant and official language in Iran) by one of the trained investigators (SZ) at different locations, chosen by the participant. The interviewer used an interview guide, which was developed for the purpose of this study. To build rapport with the participants, the interviewer (SZ) initiated the interview with small talks that were engaging and make the participants feel comfortable, respected, and feeling that the interviewer thinks along the same lines as them. To align the study questions with the study aim, we asked participants to explain, in detail, their motives for providing care to PWD. We also used several probing questions to delineate more specific and detailed answers. Each interview lasted between 45 to 60 min. In addition, we collected several basic demographic information to describe the study sample characteristics (Additional file 1).

### Data analysis

The interviews were recorded and subsequently transcribed verbatim in Farsi. The qualitative data were concurrently organized and analyzed using inductive content analysis [25, 26], in which theory is derived from data and then demonstrated by characteristic examples of data [21]. We used “Coding Consensus, Co-occurrence, and Comparison” methodology [27], which involved reviewers (SZ, ME) independently reading and rereading the initial verbatim transcript, identifying key sub-themes according to the participants’ phrases, and performing initial coding. Subsequently, another member of the research team (FZA) randomly selected sections of the transcripts and independently followed the same methodology to verify identified codes, and resolve any thematic issues. This approach is recommended in qualitative research to enhance the verifiability of the data analysis [28, 29]. The final codes were then categorized according to their similarities and differences [30, 31].

### Trustworthiness

To ensure that the nuances of data are reflected in coding, different members of our research team scrutinized and evaluated the transcripts, codes, and categories. In addition, we followed the recommended and accepted methodology and relied on the qualifications and expertise of an experienced, multidisciplinary research team to collect rich data and conduct reliable analysis [25, 28, 31, 32]. The team included an experienced investigator with an extensive background in qualitative study and health education (FZA, SBH), two health educators (SZ and AE), and two psychiatrists (ME and MB). Furthermore, for publication purposes, SZ and FZA translated the quotes to English, and their translation was back-translated by SHB to ensure accuracy.

## Results

Participants consisted of 16 women and three men (total, *n* = 19) who were the informal caregivers of PWD. All the informal caregivers were among friends, wives, daughters, sons, husbands, daughters-in-law, and nieces, or nephews of PWD. Participants ranged in age from 23 to 84, with a mean age of 51.89 ± 5.62 years. The majority were married (About 84%) and literate (88%), (Table 1). None of the informal caregivers had received training regarding caring for PWD.

**Table 1:** Sociodemographic characteristics of participants

	Caregivers (*n* = 19)	
Variable	Number of Respondents	Percentages
Sex		
Female	16	84.2
Male	3	15.8
Age (years)		
18–27	1	5.3
28–37	2	10.5
38–47	4	21.1
48–57	7	36.8
58–67	3	15.8
> 68	2	10.5
Marital status		
Married	16	84.2
Single	3	15.8
Education Level		
No formal education	2	10.5
Less than 12 years	2	10.5
High School Diploma	8	42.1
Some University/Graduate	7	36.9
Relationship to patient		
Wife	4	21.1
Daughter	9	47.4
Son	1	5.2
husband	1	5.2
Nephew and Niece	1	5.2
Daughter-in-law	2	10.5
Neighbor and or Friend	1	5.2

We identified four categories of psychosocial motives based on informal caregivers’ feedback and experiences. These include in the order of presentation 1) Moral-based motives, 2) Religious, and spiritual motives; 3) Financial motives, and 4) Wicked motives (Table 2).

**Table 2:** The caregivers’ motives for caring for the PWD

Main Categories	***Subcategory***
*1. Moral-Based Motives*	*1.1. Satisfying one’s moral principle and sense of commitment and compassion to the family*
	*1.2. Nurturing devotion and selflessness*
2. Religious and Spiritual Motives	*2.1. Adherence to religious beliefs and inscription*
	*2.2. To receive spiritual or divine reward*
	*2.3. To avoid divine punishment*
	*2.4. Other religious beliefs and associations*
2.3. Financial Motives	*3.1. Attaining living accommodations*
	*3.2. Gaining inheritance entitlement*
4.Wicked Motives	*4.1. Getting revenge*
	*4.2. Stealing*

### Moral-based motives

#### Satisfying one’s moral principle and sense of commitment and compassion to the family

Children of the PWD, who served as the informal caregivers, considered taking care of their parents an honorable duty and believed that they were morally obligated to take care of them in old age, regardless of their health status. A 40–60-year-old participant stated, “*Besides the inner satisfaction and contentment that taking care of my mother gives me, I consider it my moral duty to serve my mother.”*

Several spouses of the PWD considered taking care of their husbands or wives a way of acknowledging their commitment to a lifelong love and care they shared as couples. One caregiver said: “*For our love and for a lifetime of romantic life together (crying), I am committed to taking care of my spouse.”* Another informal caregiver put it this way: “*It is hard, I feel like I have lost my romantic and relational partner, but I do not get mad or resent this situation. I feel lonely. It is like someone has tightened one of your hands behind you and from now on you have to go along with life only with one hand. But when we married, we pledged to a lifelong commitment to each other. Only someone who is in my position could fully understand what I mean.”*

A caregiver of a PWD expressed: “*My grandmother cultivated compassion and empathy in our family by way of taking care of my grandfather, despite her old age. In a way, she cared about him more than herself. This is a reason my siblings and I share taking care of our mother. This is our way of showing our commitment to the values that were nurtured in our family.*

An informal caregiver mentioned that “*My husband’s mental health has been deteriorating. I was offered to place him in a nursing facility/home. I decided against it. Although I’m old and it’s difficult for me to take care of him, but I think he is safer and happier at home. This is the least I can do (… He is still the love of my life.) “*. This feeling of giving of oneself to facilitate the well-being of a patient was common among family members who cared for PWD.

#### Nurturing devotion and selflessness

According to *one of the caregivers*, the thought of getting diagnosed with ‘Alzheimer’s is a troubling thought that comes to mind as one gets older and requires dedication and selflessness from the side of the family. Following is part of her statement: *“I have a toxic in-law. She is very self-centered, spiteful, stubborn, and controlling. It is easy for her to hurt whoever gets close to her. Her children are annoyed with her and do not want to stay and take care of her. But my approach is to nurture dedication and selflessness in my children. My husband and I, along with our children, visit her almost every day. I cook, feed, and bathe her. Children help with cleaning the house, and my husband helps with shopping and grocery. Hopefully, I can nurture the spirit of selflessness in my children.”*

### Religious and spiritual motives

#### Adherence to religious beliefs and inscription

Several other caregivers mentioned obeying God’s commands as reflected in the Quran (Muslim Holy Book) inscriptions as the main reason they took care of a family PWD. One middle-aged caregiver said, *“This disease has been mentioned in Surah Al-Hajj, verse 5, and there are also verses in the Quran about the need to take care of parents in old age or generally older people.” (*Surah Al-Isra*, verses 23 and 24).*

#### To receive spiritual or divine reward

The participants believed that they would be spiritually rewarded for the honest care they provide to PWD. One participant articulated this notion as the following: *” I have noticed how serving my mother has positively affected my life. I had a bad car accident a while back. From the look of my car, everybody thought the driver must have been dead. After the accident, I experienced numbness and tingling* in my spine*, indicating the possibility of becoming paralyzed. However, as you see, I am OK. I truly believe it was my mother’s prayers that helped me to recover.”* (A 30–40-year-old caregiver).

#### To avoid divine punishment

The motivation for salvation from divine retribution was also one of the factors associated with caring for sick family members. One participant said: “*My father was diagnosed with Alzheimer’s last year. His behavior does no longer resembles the kind man he was. It is hurtful the way he treats us. But we take care of him in spite of this. We believe God observes will condemn us if we do not take care of him. The scriptures in the Quran inspires the worshippers to be kind and compassionate to the sick.”* (A middle-aged caregiver).

#### Other religious beliefs and associations

For some participants, certain religious beliefs and associations provided the incentive and motivation for caring for an older adult with Alzheimer’s. A middle-aged caregiver explained that “*My mother and I are taking care of a patient who has Alzheimer’s disease because she is a descendant of the Prophet Mohammad (a* Saadat*). In Muslin religion it is considered as an honor to serve Prophet Mohammad descendants …*. *It probably would have been different if she was not a Saadat. We serve this patient wholeheartedly.”*

### Financial motives

#### Attaining living accommodations

The need for a place to live was another motivating factor. *“... my husband was.sick. We spent everything we had to take care of his needs, but he died two years ago. Since my baby and I didn’t have a place to live, we moved to one of my relatives’ house. She has Alzheimer’s disease. On days, when her daughter is at work, I take care of her. I am satisfied, and they are appreciative. Her house is big, and I have a room for myself and my baby. I don’t have to look for a place to live. It would have been challenging with a baby”.* (A young caregiver).

#### Gaining inheritance entitlement

Sometimes family members of the Alzheimer’s patient informally and in private arrangements offer a share of the patient’s inheritance to the caregiver as an incentive. This unspoken rule is motivating, as stated by the following participant: *“My mother was diagnosed with Alzheimer’s disease nearly three years ago. At that time, my sister and brother lived out of the state and couldn’t provide care for her. Instead, they offered me my mother’s estate, which was her only asset. They motivated me to live with my mother and take care of her by way of waving their share of the inheritance. My mother is dear to all of us, but they cannot live here. It comforted them when I accepted the offer. They come to see my mother two or three times a year.”* (An over forty years old caregiver).

### Wicked motives

#### Getting revenge

Of the participants, we found those who aimed to settle their hostility to the family at the expense of the care receiver’s’ health. A caregiver explained, “*I have been living with my husband’s family for years, and I’ve been hurt a lot by them. I forgave them for the sake of my children. But now they are making me to take care of my father-in-law. It is not fair that they have put me in this position. He has Alzheimer’s. So I just make sure he is fed well. That is all. Other needs, I neglect. This is my way of getting revenge for my in-law’s abusive behaviors towards me, and my family.*” (An over forty years old caregiver).

#### Stealing

A caregiver of an Alzheimer’s patient stated that my father’s family is looking for an opportunity to take monetary advantage of his situation. This participant explained: “*Knowing of my father’s dementia, a couple of relatives came to our house to help by way of taking him out to a park. However, later on, I found out that they had taken him to a public notary and made him sign away some of his properties in their name. I tried hard to prove that my sick father was incapable of making such decisions. Just recently, finally, I was able to prove their crime through legal action.”* (An adult caregiver).

## Discussion

In this qualitative study, the general underlying motives of the informal caregivers of PWD were: 1) Following a moral principle and nurturing them; 2) Having respect for religious scripture, and obeying one’s religious and spiritual beliefs; 3) Gaining financial advantage, and 4) Bing mean and immoral.

Perceiving that caring for PWD is morally justified, and religiously and spiritually fulfilling, aligns with the Iranian tradition and Islamic belief [2]. It encourages family support, respect for the elders, and altruistic respect for patients, which is ultimately self-satisfying [33, 34]. Religiosity has been linked to having an agreeable and conscientious attribute [35]. It has been suggested that having a satisfying experience with caregiving responsibilities reduces caregiver stress, helps with coping [36], and enhances the quality of care [37]. In Iran, PWD, often, is cared for by their wives and/or daughters [38]. Motivated morally, religiously, and spiritually, they are poised to provide ideal care for these individuals, one that is based on a loving, kind, and understanding attitude, commitment, compassion, and kindness toward the care receiver [15, 39, 40].

A few informal caregivers expressed pecuniary incentives as a means to reinforce and boost their motivation in providing care for a PWD. However, not the primary motivation. As reported in the previous studies, employment and financial gain are significant motivators for formal caregivers in caring for PWD [6, 41–43].

The finding that a few participants were also driven by an inner desire to financially abuse the caregiving situation, points to the need for strategies that safeguard PWD against susceptibility to financial exploitation. In a previous study, of a total of 125 elderly who were interviewed, 9.6% reported experiencing abuse including neglect, physical or financial abuse. However, elderly patients are reluctant to report abuse [44]. In cases where providing care by an informal caregiver is the option, the level of safeguard could be aligned with the patient’s level of vulnerability, nature of relationship between the caregiver and the care receiver, benefits gained by the caregiver, and the level of influence the caregiver has on the care receiver to commit wrongdoing [45]. Several screening tools are available to identify caregivers’ wrongdoings, but more research is needed to establish their validity and applications for caregivers of PWD [46, 47].

### Study limitations

Our results have limited reproducibility since this is a qualitative study, but offer some insights into the research that can be built upon in further studies. Other controversies around qualitative studies have to do with the trustworthiness of results [48]. We have considered the following to enhance the trustworthiness of our findings. We utilized different types of informal caregivers to capture different views and experiences of the participating caregivers. We performed a rigorous data collection and comprehensive data analysis to support data saturation and robust reports of data. And, we corroborated the results by using multiple reviewers to ensure that participants’ viewpoints were adequately interpreted. Nonetheless, the extent to which our findings could be generalized to other cultures, especially those which are secular, needs further investigation.

## Conclusions

Our results identified several aspects of motivation for providing care to PWD by the informal caregiver. They include moral, religious, and spiritual aspects; sharing housing accommodations, and the likelihood of inheriting a portion of the patient’s assets based on unspoken rules and informal arrangements in the family, and wicked and immoral aspects. These findings can inform future investigators who aim to enhance the experiences of informal caregivers of PWD, and subsequently, the quality of the care they provide. It is also important to study how caregivers’ motivations change as their responsibilities evolve. Moreover, exploratory research into factors, structures, and processes through which informal caregivers can provide optimal care to PWD should be encouraged and supported.

## Supplementary information

**Additional file 1.** Study demographic questionnaire.

## Data Availability

Data used for this manuscript will be available upon reasonable request (i.e., no personal identifying information can be shared) by the corresponding author of this manuscript.

## Abbreviations

PWD: People with Dementia
