# Supplementary material for: What motivates informal caregivers of people with dementia (PWD): a qualitative study
Source: BMC Palliat Care. 2019 Nov 28;18:105. doi: 10.1186/s12904-019-0491-9 (PMC6883577; doi:10.1186/s12904-019-0491-9)
Supplement: Supplementary file 1 — Additional file 1. Study demographic questionnaire. [file 12904_2019_491_MOESM1_ESM.docx]

**Caregiver Demographic Questionnaire**

**Dear caregiver,**

The following questionnaire is designed for conducting an academic research project and is completely confidential. Certainly, your valuable comments will help you improve the questionnaire, implementation of the project and conclusion. Therefore, answer the questions by writing the answer or selecting one of the options by inking × sign in the box in front of the option.

1) Gender: Male □ Female □

2) Age: -------- years

3) Education: High school dropout □ High school diploma□ Higher education □

4) Marital status: single □ Married □ Widow □ Divorced □

5) What is your relationship with the patient?

□ No relationship

□ Spouse

□ Child (daughter)

□ Child (son)

□ Daughter-in-law

□ Son-in-law

□ Distant relative

□ Friend

□ Granddaughter

□ Grandson

6) Do you get paid for patient care? Yes □ No □
